# Supplementary material for: Ultrathin Acoustic Parity-Time Symmetric Metasurface Cloak
Source: Research (Wash D C). 2019 Aug 19;2019:8345683. doi: 10.34133/2019/8345683 (PMC6750042; doi:10.34133/2019/8345683)
Supplement: Supplementary Materials — (1) Surface impedance derivation. Figure S1: geometry of the metasurface cloak. (2) PT symmetry field. (3) One-way cloak and angular sensitivity. Figure S2: numerical simulation when insonified from the gain side. Figure S3: numerical simulation when insonified at various angles of incidence (4). Experimental implementation. Table 1: geometrical parameters of the Helmholtz resonators. Figure S4: experimental implementation. Figure S5: electrical gain circuit. [file 8345683.f1.pdf]

Supporting Material for: Ultrathin acoustic parity-time symmetric metasurface cloak

Hao-xiang Li,[1] María Rosendo-López,[2] Yi-fan Zhu,[1] Xu-dong Fan,[1]
Daniel Torrent,[3] Bin Liang,[1] Jian-chun Cheng,[1] and Johan Christensen[2]

[1]*Key Laboratory of Modern Acoustics, MOE,*
Institute of Acoustics, Department of Physics,
Collaborative Innovation Center of Advanced Microstructures,
Nanjing University, Nanjing, 210093, China

[2]*Department of Physics, Universidad Carlos III*
de Madrid, ES-28916 Leganés, Madrid, Spain

[3]*GROC, UJI, Institut de Noves Tecnologies de la Imatge (INIT),*
Universitat Jaume I, 12080 Castellè, Spain

I. SURFACE IMPEDANCE DERIVATION

Perfectly rigid cylinder

Let us assume that a harmonic incident field $P_0 = P_0(\mathbf{r}, \omega)$ impinges an acoustically thin surface located at $r = b$ surrounding a perfectly rigid cylindrical obstacle with radius $r = a$.

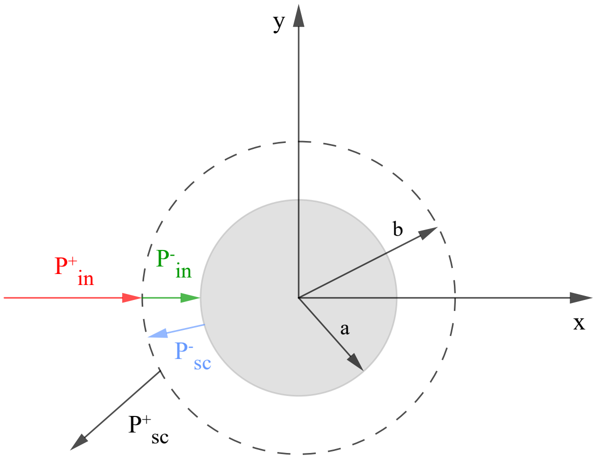

FIG. 1. Geometry of the metasurface cloak including the incoming and scattered wave components.

The aim is to derive the necessary surface impedance for which no scattering is permitted. In the region $r > b$ the total acoustic pressure reads

$$P^+ = P^+_{in} + P^+_{sc}. \tag{1}$$

In the absence of back scattering where $P^+_{sc} = 0$, the total pressure thus becomes

$$P^+(\mathbf{r}, \omega) = P_0(\mathbf{r}, \omega) = \sum_q A^0_q J_q(kr) e^{iq\theta}. \tag{2}$$

In the gap region $a \le r \le b$, sound is confined between the metasurface and the rigid obstacle at which the normal velocity has to vanish. In this event, we can write the total acoustic pressure in the following form

$$P^-(\mathbf{r}, \omega) = \sum_q B_q \Big[J_q(kr) Y'_q(ka) - J'_q(ka) Y_q(kr) \Big] e^{iq\theta}. \tag{3}$$

We are able to express the gap scattering amplitude B_q via the incoming one A^0 when imposing pressure continuity at $r = b$. In doing so, $P^-(\mathbf{r}, \omega)$ equates to

$$P^-(\mathbf{r}, \omega) = \sum_q A_q^0 J_q(kb) \frac{Y_q'(ka)J_q(kr) - J_q'(ka)Y_q(kr)}{Y_q'(ka)J_q(kb) - J_q'(ka)Y_q(kb)} e^{iq\theta}. \tag{4}$$

The jump discontinuity at the zero-thickness interface at $r = b$, leads to a surface impedance written as

$$Z_s = \frac{P^+}{v_\perp^- - v_\perp^+}, \tag{5}$$

hence, we express the respective normal components of the particle velocity $v_\perp$ as follows:

$$\begin{aligned} v_\perp^+ &= -\frac{1}{i\omega\rho}\frac{\partial P^+}{\partial r}\bigg|_{r=b} = -\frac{k}{i\omega\rho}\sum_q A_q^0 J_q'(kb)e^{iq\theta}, \\ v_\perp^- &= -\frac{1}{i\omega\rho}\frac{\partial P^-}{\partial r}\bigg|_{r=b} = -\frac{k}{i\omega\rho}\sum_q A_q^0 J_q(kb)\frac{Y_q'(ka)J_q'(kb) - J_q'(ka)Y_q'(kb)}{Y_q'(ka)J_q(kb) - J_q'(ka)Y_q(kb)}e^{iq\theta}. \end{aligned} \tag{6}$$

Conclusively, the surface impedance at the interface $r = b$ can thus be expressed as:

$$Z_s = \frac{-P_0(b, \omega)}{-\frac{k}{i\omega\rho}\sum_q A_q^0 J_q'(ka)\left[\frac{J_q(kb)Y_q'(kb) - J_q'(kb)Y_q(kb)}{Y_q'(ka)J_q(kb) - J_q'(ka)Y_q(kb)}\right]e^{iq\theta}}. \tag{7}$$

By using the Wronskian

$$J_q(kb)Y_q'(kb) - J_q'(kb)Y_q(kb) = \frac{2}{\pi kb}, \tag{8}$$

and Taylor expanding $J_q(kb)$ and $Y_q(kb)$ around a as follows

$$\begin{aligned} J_q(kb) &\approx J_q(ka) + (b-a)J_q'(ka) \\ Y_q(kb) &\approx Y_q(ka) + (b-a)Y_q'(ka), \end{aligned} \tag{9}$$

Eq. (7) simplifies to

$$Z_s = iZ_0 \frac{b}{a} \frac{P_0(b, \omega)}{\frac{\partial P_0(\mathbf{r}, \omega)}{\partial (kr)}\Big|_{r=a}}, \tag{10}$$

where the free space impedance $Z_0 = \rho_0 c_0$.

Specifically, we will examine the case of a plane wave of unitary real amplitude propagating parallel to the x-axis. Thus we can write

$$P_0(\mathbf{r},\omega) = e^{ik(r\cos\theta)}, \tag{11}$$

where θ is the angle in polar coordinates. Hence, when substituting Eq. (11) into Eq. (10) to derive the surface impedance for the example of a plane wave

$$Z_s = Z_0 \frac{b}{a} \frac{e^{ik(b-a)\cdot\cos\theta}}{\cos\theta}. \tag{12}$$

Under the assumption of a deeply subwavelengh air gap separating the ultrathin metasurface cloak from the rigid cylinder, i.e., $k(b-a) \ll 1$, we split the complex surface impedance Z_s into its real and imaginary components

$$\begin{aligned} \mathrm{Re}(Z_s) &\approx Z_0 \frac{b}{a} \frac{1}{\cos\theta} \\ \mathrm{Im}(Z_s) &\approx Z_0 \frac{b}{a}\, k(b-a). \end{aligned} \tag{13}$$

Pressure release cylinder

In the following we derive the surface admittance expression for the same scenario as above, however for a sound soft cylinder. We aim at expressing the admittance to find its equivalence to the microwave analogue system comprising TM waves irradiating a perfect electric conductor[1].

Pressure insonifying a pressure release body vanishes at its surface, $P = 0$. Hence, the total acoustic pressure in the region $a \leq r \leq b$ can be written in the following way:

$$P^{-}(\mathbf{r},\omega) = \sum_q B_q \Big[J_q(kr)Y_q(ka) - J_q(ka)Y_q(kr) \Big] e^{iq\theta}. \tag{14}$$

From Eq. (14) it can be clearly seen that the pressure release condition is fulfilled at the boundary of the soft cylinder $P^{-}(a,\omega) = 0$. As we did above, the gap scattering amplitude B_q is expressed in terms of the incoming one A_q^0 :

$$P^{-}(\mathbf{r},\omega) = \sum_q A_q^0 J_q(kb) \frac{J_q(kr)Y_q(ka) - J_q(ka)Y_q(kr)}{J_q(kb)Y_q(ka) - J_q(ka)Y_q(kb)} e^{iq\theta}. \tag{15}$$

The surface admittance at a jump discontinuity, similar to Eq. (5), is written as

$$Y_s = \frac{v_\perp^- - v_\perp^+}{P^+}, \tag{16}$$

thus the particle velocities across that interface, slightly modified compared to the rigid case, read

$$\begin{aligned} v_\perp^+ &= \frac{1}{i\omega\rho}\frac{\partial P^+}{\partial r}\bigg|_{r=b} = \frac{k}{i\omega\rho}\sum_q A_q^0 J_q'(kb) e^{iq\theta}, \\ v_\perp^- &= \frac{1}{i\omega\rho}\frac{\partial P^-}{\partial r}\bigg|_{r=b} = \frac{k}{i\omega\rho}\sum_q A_q^0 J_q(kb)\frac{J_q'(kb)Y_q(ka) - J_q(ka)Y_q'(kb)}{J_q(kb)Y_q(ka) - J_q(ka)Y_q(kb)} e^{iq\theta}. \end{aligned} \tag{17}$$

Conclusively, we can express Eq. (16) in the following form:

$$Y_s = -\frac{1}{P_0(b,\theta)}\frac{k}{i\omega\rho}\sum_q A_q^0 J_q(ka)\left[\frac{J_q(kb)Y_q'(kb) - J_q'(kb)Y_q(kb)}{J_q(kb)Y_q(ka) - J_q(ka)Y_q(kb)}\right] e^{iq\theta}. \tag{18}$$

As in the previous subsection, we employ the Wronskian, take $A_q^0 = i^q$ for a plane wave of the following form $P_0(b,\theta) = e^{ikb\cos\theta}$ and use the free-space admittance $Y_0 = \frac{k}{\omega\rho}$ to obtain

$$Y_s = iY_0 \frac{2}{\pi kb} e^{-ikb\cos\theta} \sum_q i^q \frac{J_q(ka)}{J_q(kb)Y_q(ka) - J_q(ka)Y_q(kb)} e^{iq\theta}. \tag{19}$$

Interestingly, the necessary surface admittance to remove back-scattering of an insonified soft cylinder as expressed in Eq. (19) is equivalent to the one for TM microwaves impinging a perfect conducting wire (Eq. (2) in ref. 1).

II. $\mathcal{PT}$-SYMMETRIC FIELDS

Here we are studying the $\mathcal{PT}$ symmetry properties of the sound fields surrounding the cloak. We take reference from the angular convention presented in Fig. 3(a) of the manuscript. Considering both the polar parity and time reversal operation for the pressure, we define

$$\begin{aligned} \mathcal{P}[P(\pi+\theta,t)] &= P(\theta,t), \\ \mathcal{T}[P(\pi+\theta,t)] &= P^*(\pi+\theta,-t). \end{aligned} \tag{20}$$

Explicitly we can write the expression of the pressure plane wave of complex amplitude B that impinges the loss semi-circle at $r = b$

$$P(\pi \pm \theta) = Be^{-ikb\cos(\pi\pm\theta)}. \tag{21}$$

By applying the parity inversion on the pressure, that is, transforming Eq. (21) to its mirror image in polar coordinates and further employing a time-reversal operation, we obtain the pressure at the gain semi-circle. Here we can employ either the + or the −sign in the angular position thanks to the symmetry of the geometry. We choose to derive the pressure via the surface impedance and the velocities across the jump discontinuity from Eq. (5) as follows

$$P(\theta) = \mathcal{PT}[P(\pi+\theta)] = -\mathcal{PT}[Z_s(\pi+\theta)]\Big(\mathcal{PT}[v_\perp^+(\pi+\theta)] - \mathcal{PT}[v_\perp^-(\pi+\theta)]\Big). \tag{22}$$

The individual $\mathcal{PT}$ operations in Eq. (22) result in

$$\mathcal{PT}[Z_s(\pi+\theta)] = -\frac{b}{a}\frac{Z_0}{\cos(\theta)} - kZ_0\frac{b}{a}(b-a)i = -Z_s(\pi+\theta), \tag{23}$$

while the velocities from Eq. (6), with the new angular convention however, read

$$\begin{aligned} \mathcal{PT}[v_\perp^+(\pi+\theta)] &= \frac{k}{i\omega\rho}\sum_q (A_q^0)^* J_q'(kb) e^{-iq\theta}, \\ \mathcal{PT}[v_\perp^-(\pi+\theta)] &= \frac{k}{i\omega\rho}\sum_q (A_q^0)^* J_q(kb)\frac{Y_q'(ka)J_q'(kb) - J_q'(ka)Y_q'(kb)}{Y_q'(ka)J_q(kb) - J_q'(ka)Y_q(kb)} e^{-iq\theta}. \end{aligned} \tag{24}$$

Finally, the pressure $P(\theta)$ emitting the gain metasurface in Eq. (22) is written as

$$\begin{aligned} P(\theta) &= \Big(\frac{b}{a}\frac{Z_0}{\cos(\theta)} + kZ_0\frac{b}{a}(b-a)i\Big)\left(\frac{k}{i\omega\rho}\frac{2}{\pi ka}\frac{\pi kb}{2}\mathcal{PT}\left[\frac{\partial P_0(\mathbf{r},\omega)}{\partial(kr)}\bigg|_{r=a}\right]\right) \\ &= \Big(\frac{b}{a}\frac{Z_0}{\cos(\theta)} + kZ_0\frac{b}{a}(b-a)i\Big)\Big(\frac{k}{i\omega\rho}\frac{2}{\pi ka}\frac{\pi kb}{2} i\cos\theta B^* e^{ika\cos\theta}\Big), \end{aligned} \tag{25}$$

which can be reduced to

$$P(\theta) = B^* e^{ikb\cos\theta}. \tag{26}$$

The acoustic intensities $I(\theta) = |P(\theta)|^2$ at the respective sides of the cloak, i.e., the absolute squares of Eq. (21) and Eq. (26) thus suggest a symmetric profile

$$I(\theta) = I(\pi \pm \theta). \tag{27}$$

III. ONE-WAY CLOAK AND ANGULAR SENSITIVITY

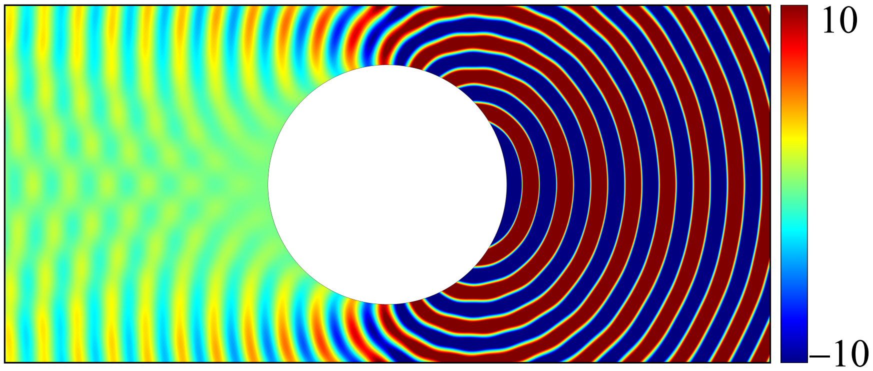

FIG. 2. Numerical simulation of the $\mathcal{PT}$ symmetry cloak when insonified from the gain side (right to left).

To confirm that our design in fact only supports one-way unhearability, we conducted simulations as depicted in Fig. 2 at which sound is incident from the opposite side producing massive back-reflection from the insonified gain semi-shell. Also, as simulations depict in Fig. 3, sound coming in toward the loss semi-shell, i.e., from left to right, yield only perfect cloaking at normal incidence. For other angles of incidence, Fig. 3 depicts strong forward scattering.

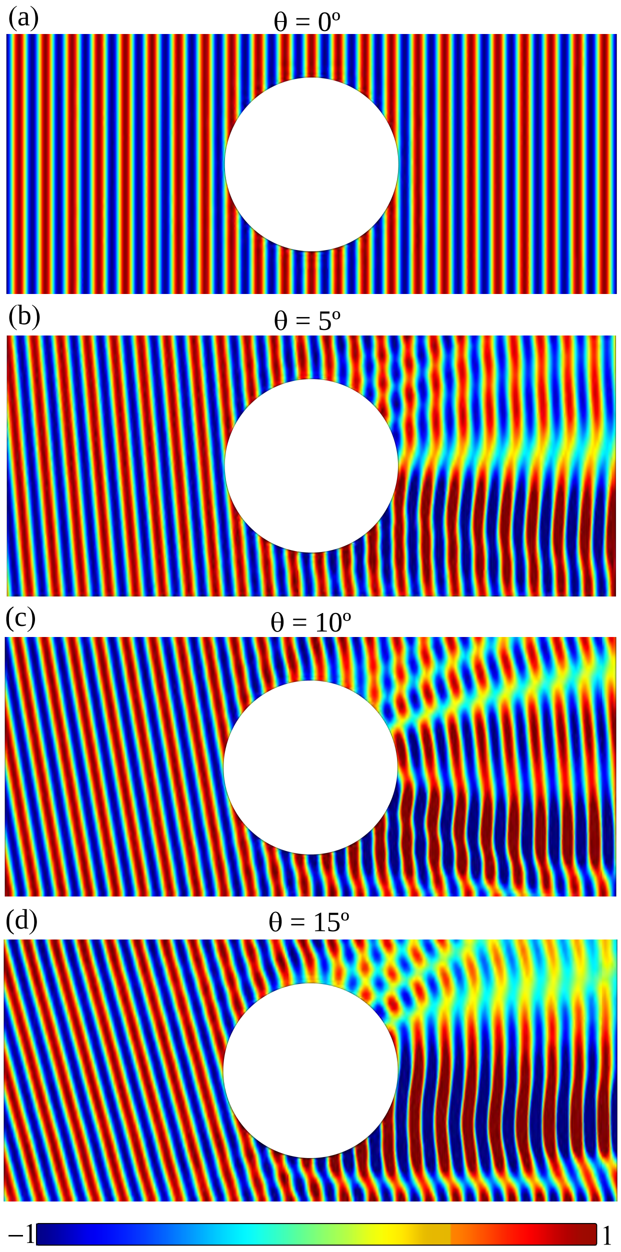

FIG. 3. Numerical simulation of the $\mathcal{PT}$ symmetry cloak when insonified from the loss side (left to right) at various angles of incidence, $\theta = 0 - 15°$.

IV. EXPERIMENTAL IMPLEMENTATION

Helmholtz resonators

In Table I the geometrical parameters for the Helmholtz resonator array are listed.

TABLE I. Geometrical parameters of the Helmholtz resonators

Element (i)	1	2	3	4	5	6	7	8
Angle (°)	0	12	24	36	48	60	72	84
w (mm)	0.08	0.08	0.08	0.08	0.08	0.06	0.06	0.06
t (mm)	0.096	0.098	0.099	0.104	0.144	0.090	0.132	0.540

Experimental setup

The experiment is carried out in a two dimensional waveguide with a uniform height of 6 cm. As can be seen in the illustration below, a line speaker array was employed to launch an

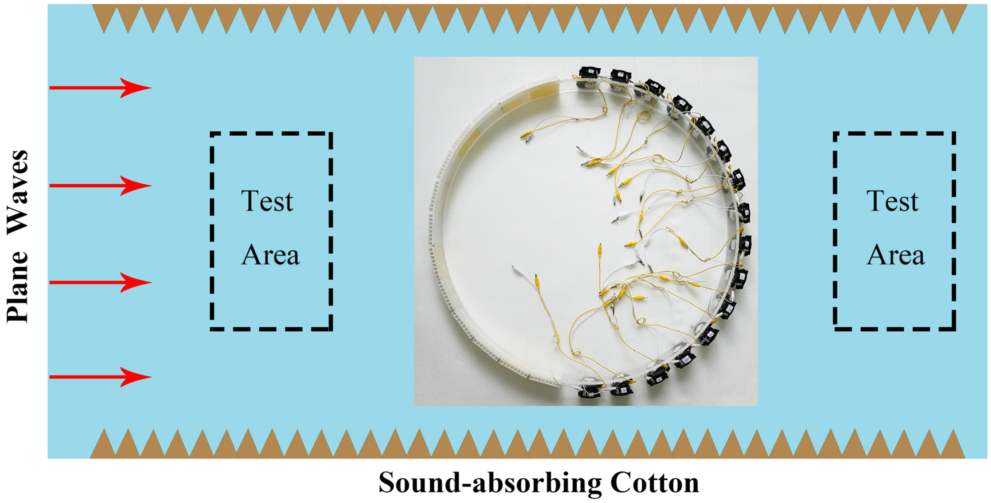

FIG. 4. Experimental implementation of the parity-time symmetric metasurface cloak.

incoming plane wave. In order to reduce unwanted reflections we covered the inner walls of the waveguide with sound-absorbing cotton. The experimental implementation is detailed in the supplementary information.

Active loudspeaker system

In this section we discuss the implementation of the active component of the metasurface cloak. In Fig. 5 we present the schematic of the circuit implemented to control the active gain component of the metasurface cloak.

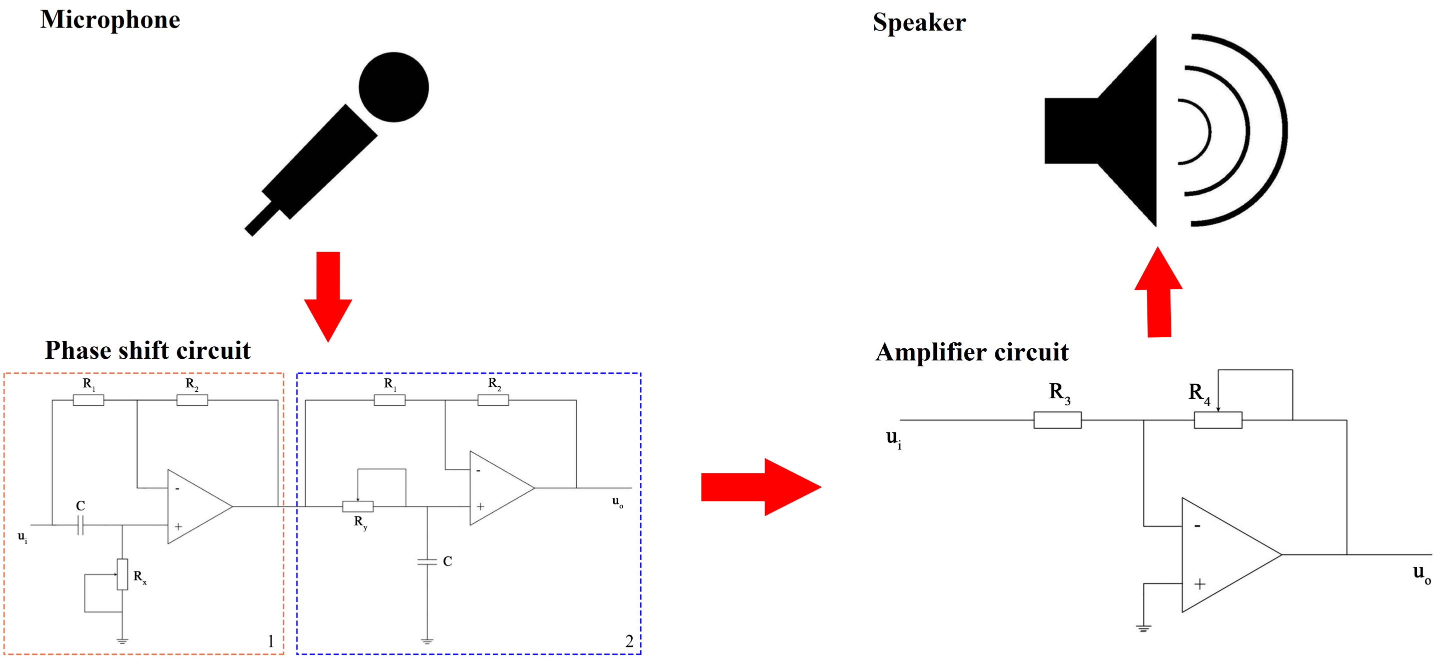

FIG. 5. Electrical circuits controlling the gain component of the metasurface cloak.

A microphone is placed in the nearest proximity to the Helmholtz resonators to record the incoming sound field. The signal is then connected to a phase shifting circuit, in which the R_x resistance controls a phase lead from the 0 to 180° and the R_y resistance controls and equivalent but lagging phase. With the right phase and increased amplitude, sound is re-emitted through the loudspeakers into the far-field.

V. BIBLIOGRAPHY

[1] Sounas, D. L., Fleury, R. & Alu, A. Unidirectional cloaking based on metasurfaces with balanced loss and gain. *Phys. Rev. Applied* **4**, 014005 (2015).
